# Phloxine B as a Probe for Entrapment in Microcrystalline Cellulose

**DOI:** 10.3390/molecules17021602

**Published:** 2012-02-07

**Authors:** Paulo Duarte, Diana P. Ferreira, Isabel Ferreira Machado, Luís Filipe Vieira Ferreira, Hernan B. Rodríguez, Enrique San Román

**Affiliations:** 1 Centro de Química-Física Molecular, and IN-Institute of Nanoscience and Nanotechnology Complexo Interdisciplinar, Instituto Superior Técnico, Universidade Técnica de Lisboa, Av. Rovisco Pais, 1049-001 Lisboa, Portugal; Email: paulo.duarte@ist.utl.pt (P.D.); diana.ferreira@ist.utl.pt (D.P.F.); ilferreiramachado@ist.utl.pt (I.F.M.); 2 Escola Superior de Tecnologia e Gestão, Instituto Politécnico de Portalegre, Lugar da Abadessa, Apt 148, 7301-901, Portalegre, Portugal; 3 INQUIMAE / DQIAyQF, Facultad de Ciencias Exactas y Naturales, UBA, Ciudad Universitaria, Pab. II, C1428EHA, Buenos Aires, Argentina; Email: hbr@qi.fcen.uba.ar

**Keywords:** phloxine B, heterogeneous systems prompt fluorescence, phosphorescence, delayed fluorescence, microenvironmental effects

## Abstract

The photophysical behaviour of phloxine B adsorbed onto microcrystalline cellulose was evaluated by reflectance spectroscopy and laser induced time-resolved luminescence in the picosecond-nanosecond and microsecond-millisecond ranges. Analysis of the absorption spectral changes with concentration points to a small tendency of the dye to aggregate in the range of concentrations under study. Prompt fluorescence, phosphorescence and delayed fluorescence spectral decays were measured at room temperature and 77 K, without the need of sample degassing because cellulose protects triplet states from oxygen quenching. In all cases, spectral changes with time and lifetime distribution analysis were consistent with the dye coexisting in two different environments: dyes tightly entrapped between polymer chains in crystalline regions of cellulose showed longer fluorescence and phosphorescence lifetimes and more energetic triplet states, while dyes adsorbed in more amorphous regions of the support showed shorter lifetimes and less energetic triplet states. This behaviour is discussed in terms of the different dye-support interactions in both kinds of adsorption sites.

## 1. Introduction

The xanthene dyes have proved to be an extraordinarily useful class of luminescent and triplet forming dyes for theoretical studies and practical applications. The interest on these probes relies on the variations in the photophysical and photochemical behaviour with the environment [1,2,3,4,5,6]. An enhancement of the rate of intersystem crossing process for triplet forming xanthene dyes is observed on going from different alcohols to water, as well as a “blue shift” in the absorption and emission spectra [1]. Among others, this family of fluorescein derivatives comprises eosin, erythrosine, rose bengal and phloxine B.

These dyes are water-soluble and employed mostly in the form of sodium salts of their sulfonic or carboxylic acids. They are anionic, and thus easily attachable to cationic groups in solid matrices and therefore, they can be applicable to all kind of natural fibers like wool, cotton and silk as well as to synthetics like polyesters, acrylic and rayon. They are also used in paints, inks, plastics and leather.

**Figure 1:**
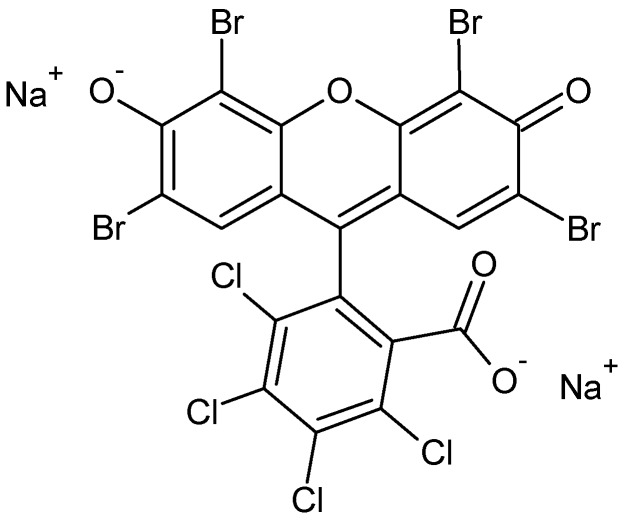
Molecular structure of Phloxine B.

Phloxine B (PhB, Figure 1), is a red acid dye that can be used for drugs and cosmetics [7], and also as photo-insecticide, because of the formation of singlet oxygen when exposed to light [8]. It differs from fluorescein by the presence of four bromine atoms at positions 2, 4, 5 and 7 of the xanthene ring and four chlorine atoms in the carboxyphenyl ring.

In these molecules the chromophore is the xanthene ring. The phenyl group is sterically hindered and cannot lie in the plane of the xanthene ring [1]. Due to the presence of the bromine atoms attached to the xanthene skeleton and the consequent increase of the intersystem crossing rate, the photochemical properties of this dye differ significantly from those of other dyes composed exclusively of light atoms, namely rhodamine, oxazine, fluorescein or acridine dyes, in which the triplet quantum yield is usually small.

Taking into account the spectroscopic properties of these dyes, several studies in solution have been reported in literature [1,2,3,4]. For erythrosin, fluorescence quantum yield (*Ф*_F_) of 0.08–0.10 and fluorescence lifetimes (τ_F_) 0.5–0.7 ns in different alcohols [1] were reported, whereas *Ф*_F_ = 0.08–0.14 and τ_F_ = 0.7−1.0 ns were determined for rose bengal in the same solvents [1]. Phosphorescence and delayed fluorescence studies of these dyes in bio-related films of starch, chitosan and gelatin were recently published [5,6].

Moreover, great interest has been focused on the assembly of dye molecules in constrained environments. This is so because the emission of organic dyes is currently enhanced when entrapped into solid matrices and because triplet forming supported dyes may be used as solid photosensitizers [9]. They may be used as environment sensors as well [10].

Literature reports several studies of dyes adsorbed on microcrystalline cellulose, such as rhodamine 6G, rhodamine 101, auramine O, rose bengal, *etc.* [11,12,13,14,15]. Adsorbed species are immobilized in the rigid cellulose matrix by a hydrogen-bonding interaction between the hydroxyl groups of the polymer chain and the guest molecule, reducing the molecular motion and increasing both the emission lifetime and quantum yield of the adsorbate, as reported in many cases. It was also reported that dyes adsorbed on cellulose are protected from quenching by molecular oxygen, since the mobility of this species is highly reduced in this medium, provided cellulose is well dried. This property is useful because triplet state properties can be studied without the interference of molecular oxygen. Small amounts of water, however, strongly affect the excited-state behaviour of the adsorbed molecule [16].

Studies of native or fibrous cellulose have shown that this medium acts as a two-phase system consisting of a less ordered and less compact amorphous region located mainly on the surface of the elementary fibrils, and well-ordered regions (crystallites) where cellulose molecules exist in a definite crystal pattern. In this latter case, cellulose molecules are aligned in a parallel fashion and closely packed together providing maximum hydrogen-bonding capacity between adjacent cellulose chains [12].

Different solvents can be used to obtained different degrees of swelling in microcrystalline cellulose, resulting in two different adsorptions of the dye: more entrapped and rigidly attached between cellulose polymer chains or adsorbed on the surface of crystallites, in a less rigid and less attached situation [12].

In this work, the absorption and luminescence properties of phloxine B adsorbed onto microcrystalline cellulose are studied. The photophysical behaviour of the dye is evaluated by reflectance spectroscopy and laser induced time-resolved luminescence, in order to characterize the state of the dye entrapped on the cellulose matrix and the effects of dye-dye and dye-support interactions on its photophysical properties.

## 2. Results and Discussion

### 2.1. Ground State Absorption Studies

**Figure 2:**
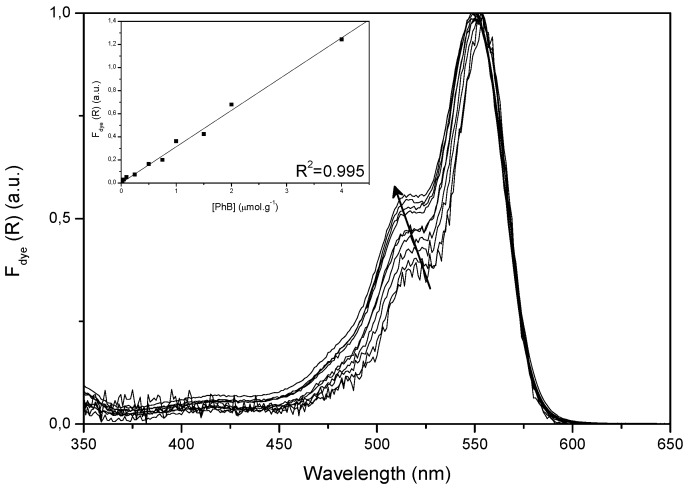
Remission function spectra of samples PhB01 to PhB10. The arrow points in direction of increasing concentration. Inset: remission function at the maximum as a function of dye concentration.

Figure 2 shows normalized remission function spectra of all samples, from which the cellulose background has been subtracted. The maximum, 551 nm, is red shifted as compared to the absorption in ethanol (547.5 nm) and in aqueous solution (538.5 nm) [17]. Absorption is rather low at wavelengths below 450 nm. The absorption of the supporting matrix (not shown in the figure) is quite broad (300–600 nm) and negligible compared with the visible absorption of the dye but becomes relevant below 400 nm. These curves evidence some dye aggregation because of the increase of the shoulder at 520 nm and the spectral broadening on increasing concentration. In spite of this, the linear fit with zero intercept of F(R)_dye_ at 551 nm as a function of dye concentration (inset) points to a reduced aggregation of the dye in the range of concentrations under study. 

### 2.2. Room-Temperature Laser Induced Luminescence

**Figure 3:**
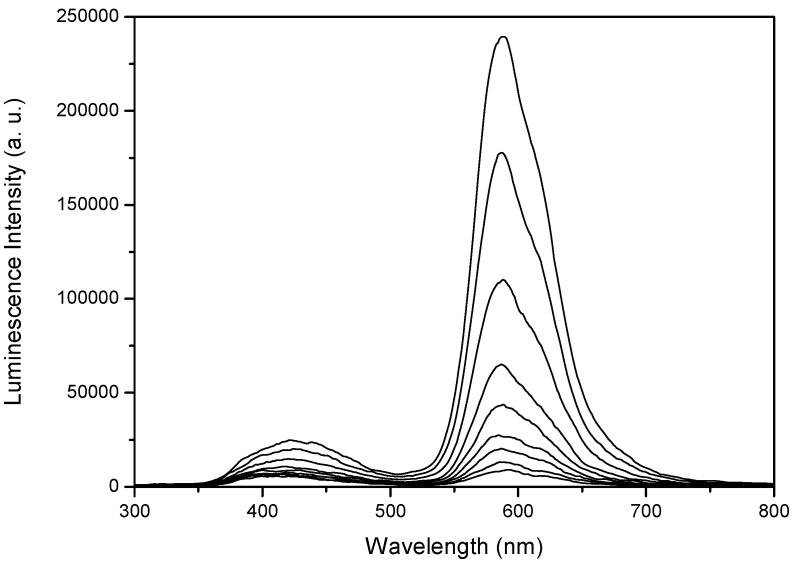
Fluorescence emission spectra for phloxine B adsorbed onto microcrystalline cellulose, 4.0 μmol g^−1^ sample, PhB10. Curves were recorded every 1 ns after the laser pulse. The excitation wavelength was 337 nm.

**Figure 4:**
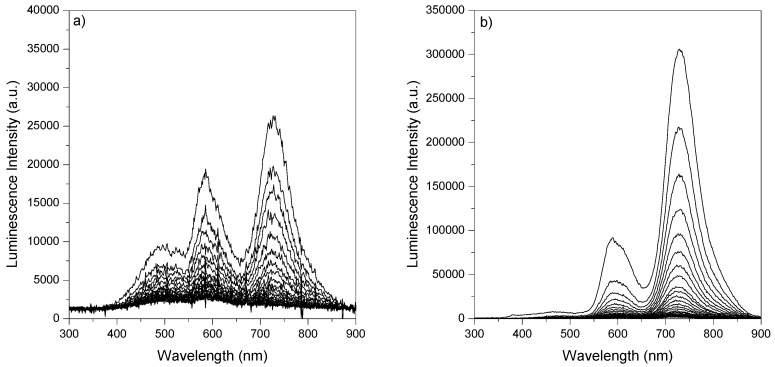
Phosphorescence and delayed fluorescence emission spectra for phloxine B adsorbed onto microcrystalline cellulose: **a)** PhB03 sample, 0.1 μmol g^−1^ (t_0_ = 100 μs, step = 100 μs); **b)** PhB10 sample, 4.0 μmol g^−1^ (t_0_ = 1 μs, step = 100 μs). The excitation wavelength was 337 nm.

Figure 3 presents the time-resolved fluorescence spectra of phloxine B adsorbed on microcrystalline cellulose (4.0 μmol g^−1^, PhB10 sample), while time-resolved emission spectra in the μs-ms range for phloxine B adsorbed on microcrystalline cellulose, with low and high loadings of the dye, are depicted in Figure 4.

As pointed out below, we used the short pulse of a nitrogen laser at 337 nm (600 ps half width) as an excitation source. This is quite suitable to time resolved emission studies in the microsecond and millisecond time range, due to its short duration, therefore avoiding deconvolution problems in the decay analysis [18]. This is not the case for studies in the picosecond-nanosecond time range, where deconvolution cannot be avoided.

The time-resolved fluorescence spectra of the sample with highest loading in the nanosecond range revealed the existence of an emission at about 590 nm, originated in phloxine B and, at shorter wavelengths, the emission from cellulose, which is dominant in the samples with low loading (spectra not shown).

Laser induced luminescence spectra in the microsecond-millisecond range were obtained with air equilibrated samples. Those recorded for argon purged samples were identical within the experimental error, both in spectral and kinetic terms. These two sets of spectra show the long lived phosphorescence of phloxine B peaking at about 729 nm and a band at about 590 nm assigned to phloxine B delayed fluorescence. This phosphorescence emission may be observed in air equilibrated samples at room temperature, since cellulose efficiently protects the triplet excited state of phloxine B from oxygen quenching, as we observed previously for many dyes and other probes adsorbed or entrapped into cellulosic chains [18,19]. Furthermore, in the spectra of the sample with low dye loading (Figure 4a), some long lived emission from cellulose can be seen at the blue side of the spectra, which is strongly reduced for highly loaded sample (Figure 4b). This reflects the fact that the fraction of photons absorbed by the substrate at the excitation wavelength is higher in sample PhB03 and reabsorption of cellulose emission increases with concentration.

**Figure 5:**
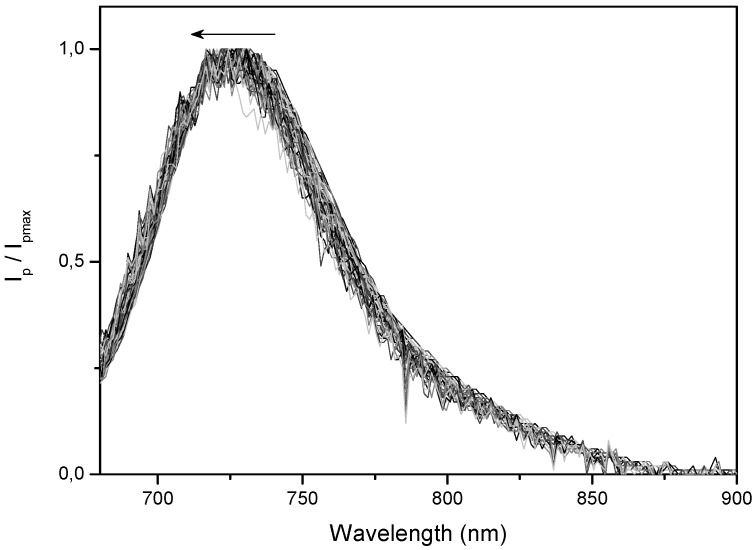
Shift of the phosphorescence spectrum observed for sample PhB10 from t = 100 μs to 6 ms in the direction of the arrow in 100 μs steps.

Phosphorescence spectra are independent of the concentration of the dye and shift to shorter wavelengths as time evolves, while keeping its shape, as shown in Figure 5 for sample PhB10. Delayed fluorescence spectra resemble direct fluorescence spectra discussed above and its shape does not change with time.

#### 2.2.1. Lifetime Distributions Analysis (LDA)

##### 2.2.1.1. Fluorescence Decay

**Figure 6:**
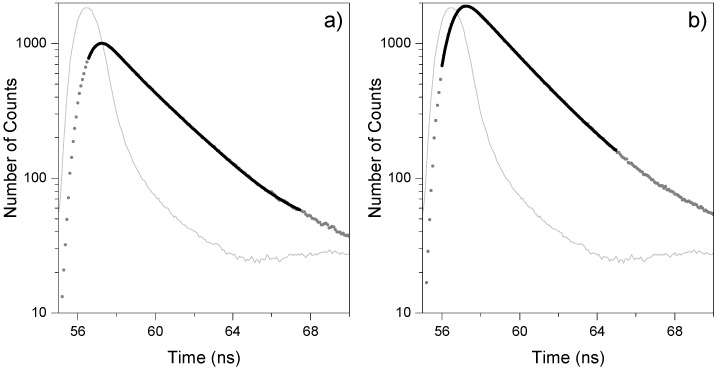
Fluorescence emission decays obtain after 310 nm excitation, **a)** for a 0.1 μmol g^−1^ sample, PhB03, while **b)** presents similar data obtained for a 4.0 μmol g^−1^ sample, PhB10.

**Figure 7:**
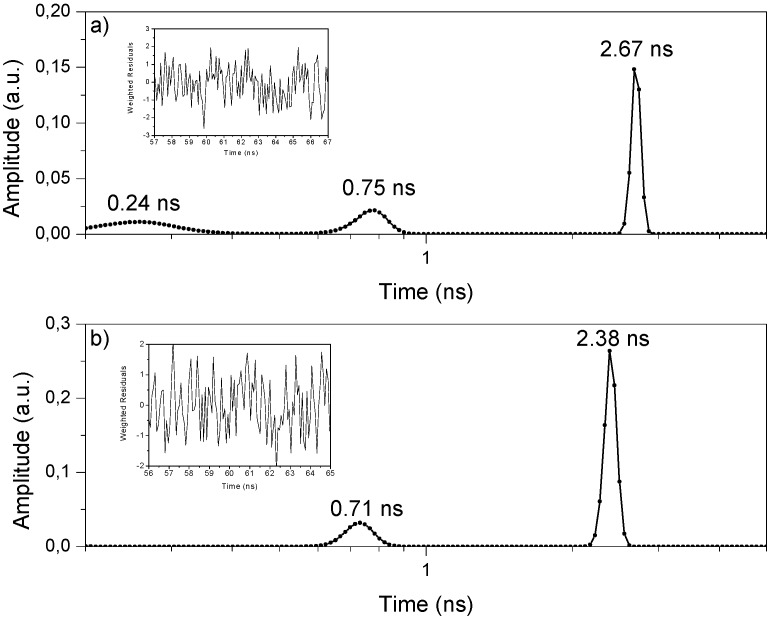
Luminescence lifetime distributions recovered from luminescence decays of air equilibrated samples: **a)** for a 0.1 μmol g^−1^ sample, while **b)** presents similar data obtained for a 4.0 μmol g^−1^ sample. Inset shows the weight residuals.

In this work, we measured fluorescence decays in the picosecond-nanosecond range using an Easylife V^TM^ equipment from OBB (see Experimental section) and analyzed the data using the software provided by the manufacturer which enables lifetime evaluation with accuracy, starting at about 90–100 ps, up to 3 μs. The fluorescence decay is approximated by a sum of exponentials with variable amplitudes. No specific profiles were used here for the determination of amplitudes, and the amplitudes and lifetimes were obtained by minimizing the chi-square function, taking into account the excitation pulse profile in the convolution matrix. Figure 6 shows the decays of samples PhB03, PhB10 and of the Ludox scattering solution (instrument response function). The fitting was done with the Exponential Series Method (ESM) and Figure 7 shows the LDA obtained for the two samples.

The results show, in sample PhB03, a very short lifetime (about 240 ps) that can be attributed to the emission of cellulose. Both samples have two different lifetimes, one short and broader at about 0.7 ns and a longer and narrower lifetime at about 2.5 ns. This indicates that the dye coexists in two different environments. The distribution at shorter lifetimes, showing the largest width, is assigned to PhB located in more disorganized parts of cellulose (more amorphous regions), in a less rigid situation, enhancing radiationless deactivation. When the dye is well entrapped into the cellulose polymer chains, a decrease of non-radiative deactivation pathways result in a largest emission lifetime. This largest lifetime is similar to that found for the same dye in polar solvents, τ ~ 3.3 ns [20], indicating that the differences in the non-radiative deactivation of the singlet excited state of the dye in the different cellulose environments are not a simple matter of rigidity of the environment, but probably includes specific interactions of the dye with the solid support due to the different availability of hydrogen bonding in the adsorption sites [21]. One possible pathway for deactivation of the excited singlet state in this media may be hydrogen bonding-assisted internal conversion [21]. This mechanism involves the conversion of the lowest vibronic level of S_1_ to a higher vibronic level of the ground state, which is able to rapidly relax assisted by hydrogen bond interactions with the solid, discharging the excess of vibrational energy to the matrix. The same mechanism may also be operative for dyes adsorbed in more amorphous regions of cellulose, where more hydrogen bonding interactions are stereochemically available. 

Yoshihara and co-workers [22] found similar results for different xanthene dyes adsorbed on different solid supports. In those cases, the shorter lifetimes were attributed to dyes adsorbed on distorted adsorption sites where internal conversion of the dye is enhanced. In these distorted sites, specific dye-support interactions exert asymmetric forces to the molecule inducing a butterfly motion at the excited state between the two halves of the xanthene ring along the short axis, which opens a new pathway for non-radiative deactivation of the excited state. In our case, PhB adsorbed in the more disorganized parts of the cellulose, where more hydrogen bonding interactions are stereochemically available, is probably able to do this butterfly flip enhancing deactivation through internal conversion and leading to shorter lifetimes. This mechanism is probably not effective for dyes adsorbed in the crystalline regions of cellulose because they are rigidly entrapped and present a lower interaction with the matrix through hydrogen bonding. However, for dyes in the more amorphous regions of cellulose, it is probably acting in conjunction with the hydrogen bonding-assisted internal conversion mechanism, leading to shorter lifetimes, almost one order of magnitude lower than the value found in solution. 

##### 2.2.1.2. Phosphorescence Decay

We developed a new tool for lifetime distributions analysis (LDA) of emissions of probes adsorbed onto heterogeneous surfaces [23]. This new methodology uses pseudo-Voigt profiles (Gaussian-Lorentzian product) instead of pure Gaussian or Lorentzian distributions and allows for asymmetric distributions. Microsoft Excel Solver was the tool used for the fitting procedures. This represents a very convenient way to treat the emission or transient absorption decay data because it reflects the multiplicity of sites available for the probe onto the specific surface under study. The use of a sum of several exponentials to analyze the decay of probes onto heterogeneous surfaces is a description without physical meaning [23]. LDA is a much better tool to study decays of a probe on heterogeneous surfaces. In this model, it is assumed that the lifetime distribution of an excited probe adsorbed on a heterogeneous, porous substrate is a consequence of a distribution of Δ*G*_0_ for the probe adsorption on the substrate around a mean value. This model allows to obtain a distribution of lifetimes, α, the relative weight in the total distribution, which reflects the decay of the emissive species in different adsorption sites. For a rigorous definition of α see reference [23].

However, the problem of recovering the distribution of lifetimes, α, from the decay curve is an inverse problem like many others in physical science, and its ill-conditioned nature is well-known and largely discussed in the literature. Therefore the validation of its conclusions should be simultaneously sustained by other spectroscopic studies.

**Figure 8:**
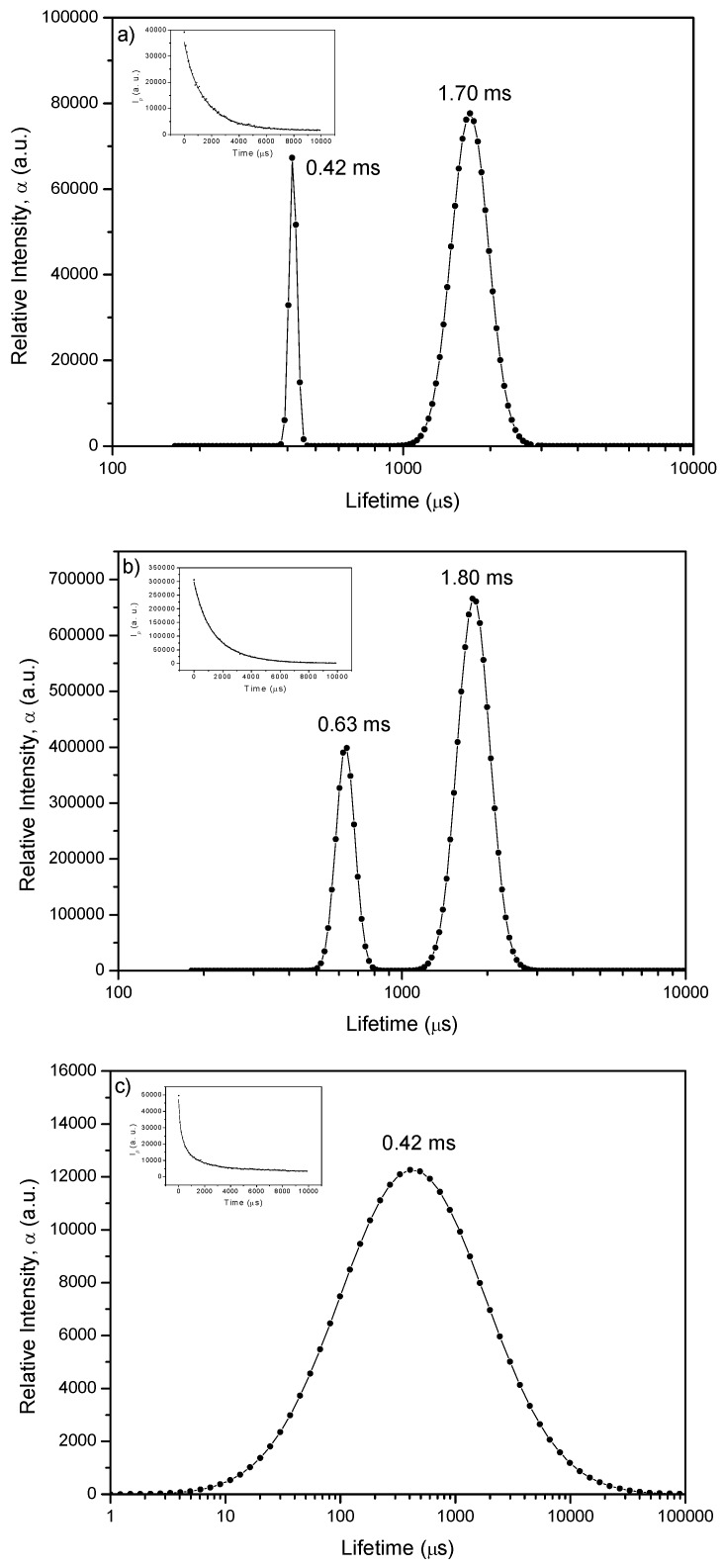
Lifetime distributions recovered from luminescence decays observed at 729 nm for: (**a**) sample PhB03 phosphorescence; (**b**) sample PhB10 phosphorescence; (**c**) Refers to luminescence of the support (microcrystalline cellulose) observed at ~ 450 nm. The insets show the fitting of the recovered decay superimposed to the experimental data.

Figure 8 shows the LDA of the phosphorescence decay for the case of phloxine B adsorbed onto microcrystalline cellulose (low loading, PhB03 sample and high loading, PhB10 sample), as well as that of the support (microcrystalline cellulose).

The PhB10 sample (Figure 8b) exhibits a dual distribution with maxima located at about 630 μs and 1,800 μs. Similar distribution was found for PhB03 sample, with maxima peaking approximately at 420 and 1,700 μs (Figure 8a). The shorter lifetime found for PhB03 has a similar value compared to the lifetime found for cellulose emission. However, this value found for PhB03 is phosphorescence of the dye and cannot be attributed to emission of the support because at the wavelength used to analyze dye phosphorescence, emission of the support is negligible. These results suggest that the dye is emitting in two different environments: one very much ordered, where phloxine is well entrapped into the cellulose polymer chains, previously swelled by the use of a protic and polar solvent, ethanol in this case [16]. In this environment, phloxine exhibits the largest phosphorescence lifetime due to the high constrain imposed by the entrapment, resulting in the decrease of the non-radiative pathways of deactivation. These lifetimes are in the order of those found in the literature for room-temperature phosphorescence of phloxine B in dye- doped PVP polymer [24]. 

In contrast with the slower component, the faster decay points to a more flexible environment. Therefore the distribution at shorter lifetimes is assigned to phloxine B located in more disorganized, *i.e.*, more amorphous regions of cellulose. In this less constrained environment, adsorption sites are characterized by interactions with stereochemically available cellulose hydroxyl groups, lowering the triplet energy and enhancing radiationless deactivation of the excited state through dissipation of vibrational energy into the matrix, leading to shorter lifetimes.

Taking into account the observed blue shift (Figure 5), the slow decaying component has to be associated with a triplet characterized by a higher energy.

### 2.3. Laser Induced Luminescence at 77 K for Phloxine B Adsorbed onto Microcrystalline Cellulose

**Figure 9:**
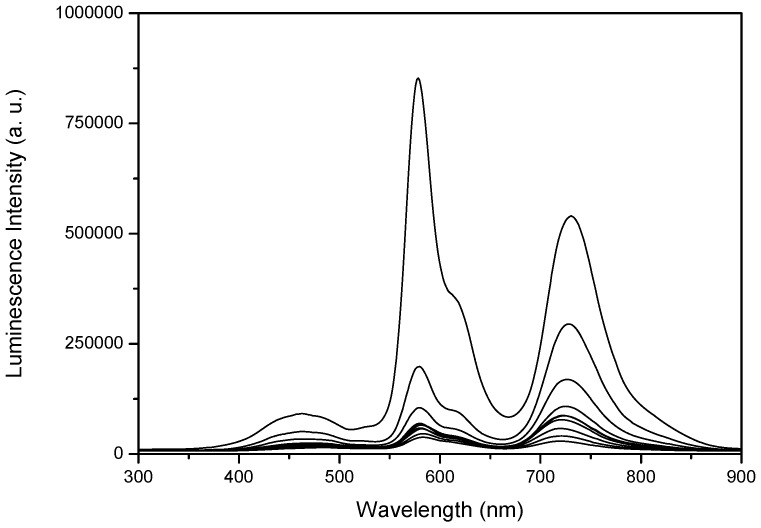
Time-resolved luminescence spectra at 77 K for phloxine B adsorbed onto microcrystalline cellulose, 4.0 μmol g^−1^ sample. Curves are separated by 1000 μs steps and curve 1 was obtained 1 μs after the laser pulse. The excitation wavelength was 337 nm.

Time-resolved luminescence spectra at 77 K (Figure 9) show again, for the long wavelength band, a decay in the millisecond time range and a blue shift of the emission spectrum with time.

The comparison between the delayed fluorescence spectra obtained at room temperature (Figure 4b) and the low temperature emission reveal that the emission peaking at about 590 nm is still present. Although back intersystem crossing is strongly reduced at 77 K, this emission is certainly derived from the dye excited by an energy transfer process from the prompt emission of microcrystalline cellulose. The delayed fluorescence spectra are superimposed at the blue side with emission from cellulose which partially overlaps the absorption of phloxine B and, therefore, part of the cellulose emission is converted into phloxine fluorescence. This fact is quite evident for lower phloxine concentrations, because cellulose prompt emission is strongly increased at 77 K, when compared to room temperature emission. 

It is interesting to note that room temperature and 77 K phosphorescence emissions of phloxine B decay with very similar rates, showing again the importance of the dye rigid environment due to cellulose entrapment. Moreover, these similarities also indicate that the different photophysical behaviour of the dye in both kinds of adsorption sites cannot be ascribed to dynamic changes of the environment during the triplet state lifetime, and may be attributed to spatial heterogeneity in the cellulose matrix.

### 2.4. Delayed Fluorescence Decay

**Figure 10:**
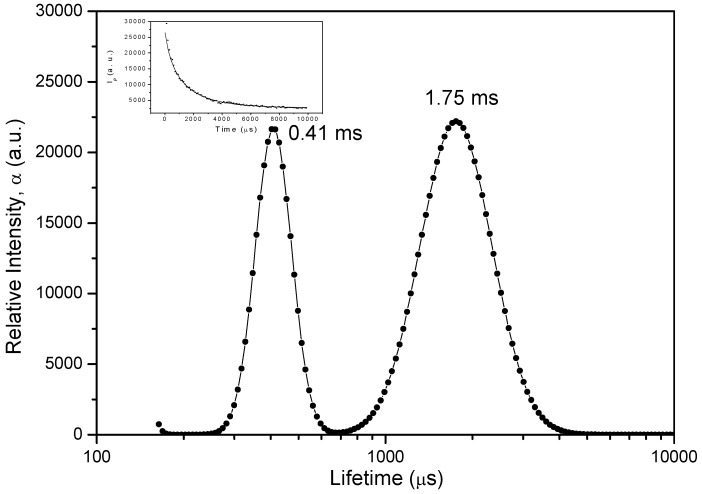
Lifetime distribution analysis of delayed fluorescence for sample PhB03, recovered from decay observed at 580 nm.

In spite of reverse intersystem crossing and energy transfer from cellulose, the lifetime distribution for the delayed fluorescence, at room temperature, provides two maxima for sample PhB03, as can be seen in Figure 10. 

The band centered at longer time has approximately the same lifetime as the longer component of the phosphorescence, 1750 μs, while the band at shorter lifetime, at about 410 μs, is quite similar to the observed lifetime of the long lived emission of pure cellulose (Figure 8c). This result seems to confirm that the band at shorter lifetime can be assigned to fluorescence resulting from reabsorption of cellulose emission. Although not resolved by LDA, the contribution to phloxine delayed fluorescence arising from fast decaying triplets might be present.

## 3. Experimental

### 3.1. Materials and Sample Preparation

Phloxine B disodium salt (PhB) was obtained from Sigma-Aldrich and used as received. Dye purity (90%) was checked by absorption spectroscopy. Microcrystalline cellulose powder (pH 5–7, average particle size 20 μm) from Fluka DSO was dried in a vacuum oven at 40 °C for 48 h before preparing the samples. Ethanol Riedel-De Haen (analytical grade) was used without further purification. The samples were prepared using the solvent evaporation method, which consists in the addition of a solution containing the probe to the powdered solid support, followed by mixing and evaporation of the solvent. The supports were dried overnight under vacuum (~10^−3^ mbar). The mixture was magnetically stirred for at least 24 h, for solvent evaporation. The final solvent removal was performed under vacuum (~10^−3^ mbar), for about 15 minutes. Several samples containing phloxine B on microcrystalline cellulose at different concentrations spanning two orders of magnitude were prepared: 0.025, 0.05, 0.10, 0.25, 0.50, 0.75, 1.0, 1.5, 2.0 and 4.0 μmol g^−1^ (samples PhB01 to PhB10).

Another set of samples were prepared by rapid evaporation of the solvent in a rotavap system (evaporation time ~20 min.). Even when these samples present more signs of dye aggregation on absorption spectra, no significant differences where observed on its photophysical behaviour compared with the slow evaporated samples. For that reason, the results of the slow evaporated samples are presented in this paper. 

### 3.2. Ground-State Diffuse Reflectance Absorption Studies

Ground-state absorption studies of phloxine B (PhB) adsorbed on microcrystalline cellulose were performed using an OLIS 14-VIS-NIR spectroscopy operating system with a diffuse reflectance attachment (90 mm diameter integrating sphere, internally coated with MgO). This apparatus has been modified to extend the initial operational range in the UV and visible region by installing a new detector (Hamamatsu R955 model, with a spectral response in the 160–950 nm range) and also to include the possibility of using short-wavelength-pass filters to prevent the fluorescence of the dyes from reaching the detector. In this case we used Comar 610 GK 50 [10]. MgO was used as a reference to adjust the 100% reflectance level. Remission function F(R) was obtained using the Kubelka–Munk equation for optically thick samples, as F(R) = (1 − R)^2^ / 2 R. Details regarding the data treatment can be found in [25] and references quoted therein.

### 3.3. Laser Induced Luminescence (LIL) Setup

A description of the LIL system was presented recently in reference [26]. For the laser-induced luminescence experiments, a N_2_ laser (PTI model 2000, ca. 600 ps fwhm, 337.1 nm excitation, ~1.0 mJ/ pulse) and a reflection geometry mode were employed. The light arising from the solid powdered samples after excitation by the laser pulse was collected by a collimating beam probe coupled to an optical fiber (fused silica) and detected by a gated intensified charge coupled device (Andor ICCD detector, i-Star 720). The ICCD was coupled to a fixed imaging compact spectrograph (Shemrock 163).

The system could be used either by capturing all light emitted by the sample (as in steady-state fluorescence spectra), or in a time-resolved mode by the use of the internal delay capability of the i-Star 720. The ICCD has high-speed gating electronics (2.3 ns) and intensifier and covers the 200–950 nm wavelength range. Time-resolved emission spectra were available in the nanosecond to second time range both in transmission or diffuse reflectance modes. The combined use of the variable time gate width and start delay facilities of the ICCD, enables one to separate prompt from delayed emissions and therefore fluorescence and phosphorescence spectra are easily available.

### 3.4. Fluorescence Lifetime Set-up

Fluorescence lifetimes were determined using Easylife V^TM^ equipment from OBB (lifetime range from 100 ps to 3 μs). This technique uses pulsed light sources from different LEDs (310 nm in this case) and measures fluorescence intensity at different time delays after the excitation pulse. In this case, 550 nm cut-off filter was used at emission both for solution and for solid samples, depending on the sample under study. The instrument response function was measured using a Ludox scattering solution. FelixGX software from OBB was used for fitting and analysis of the decay dynamics, 1 to 4 exponentials and also a lifetime distribution analysis, the Exponential Series Method (ESM).

## 4. Conclusions

Spectroscopic data shows that phloxine B excited states are sensitive to molecular environment. In microcrystalline cellulose, two kinds of environments could be sensed through the decay analysis of the singlet and triplet excited states of the dye. When the dye is tightly entrapped between cellulose chains in the crystalline regions of the support, it behaves similar to what is found in solution. This means that specific interactions between the dye and the solid support do not affect in an extreme way its photophysical behaviour compared to solution and that the dye is rigid enough not to be extremely affected by the crystalline environment. This is expected in this kind of adsorptions sites because hydrogen bonding maximizes the interactions between polymer chains, being less available to interact with the entrapped dye. 

On the other hand, dyes adsorbed in more amorphous regions of cellulose have more stereochemically available hydrogen bonding interactions with the support. These specific dye-support interactions enhance radiationless deactivation of the singlet and triplet excited states reducing their lifetimes, lowering also the energy of the triplet excited state. In the case of the singlet excited state, the lower lifetimes can be ascribed to both, a hydrogen bonding-assisted internal conversion mechanism and also an enhanced internal conversion through butterfly-like motions of the xanthene skeleton induced by hydrogen bonding exerting asymmetric forces to the molecule. Hydrogen bonding interactions also stabilize the triplet state of the dye in the amorphous regions of cellulose and facilitate non-radiative deactivation of this state through dissipation of vibrational energy into the matrix, leading to shorter triplet lifetimes.

In conclusion, phloxine B can act as a probe of spatial heterogeneity on cellulose, both through the singlet and the triplet excited states, and this sensing activity can be correlated with the specific dye-support interactions in the different adsorption sites. 
